# Cluster Analysis and Classification Model of Nutritional Anemia Associated Risk Factors Among Palestinian Schoolchildren, 2014

**DOI:** 10.3389/fnut.2022.838937

**Published:** 2022-05-10

**Authors:** Radwan Qasrawi, Diala Abu Al-Halawa

**Affiliations:** ^1^Department of Computer Science, Al-Quds University, Jerusalem, Palestine; ^2^Department of Computer Engineering, Istinye University, Istanbul, Turkey; ^3^Medical Faculty, Al-Quds University, Jerusalem, Palestine

**Keywords:** nutritional anemia, anemia classification, cluster analysis, classification model, Classification and Regression Tree, schoolchildren

## Abstract

Nutritional inadequacy has been a major health problem worldwide. One of the many health problems that result from it is anemia. Anemia is considered a health concern among all ages, particularly children, as it has been associated with cognitive and developmental delays. Researchers have investigated the association between nutritional deficiencies and anemia through various methods. As novel analytical methods are needed to ascertain the association and reveal indirect ones, we aimed to classify nutritional anemia using the cluster analysis approach. In this study, we included 4,762 students aged between 10 and 17 years attending public and UNRWA schools in the West Bank. Students' 24-h food recall and blood sample data were collected for nutrient intake and hemoglobin analysis. The K-means cluster analysis was used to cluster the hemoglobin levels into two groups. Vitamin B12, folate, and iron intakes were used as the indicators of nutrient intake associated with anemia and were classified as per the Recommended Dietary Allowance (RDA) values. We applied the Classification and Regression Tree (CRT) model for studying the association between hemoglobin clusters and vitamin B12, folate, and iron intakes, sociodemographic variables, and health-related risk factors, accounting for grade and age. Results indicated that 46.4% of the students were classified into the low hemoglobin cluster, and 60.7, 72.5, and 30.3% of vitamin B12, folate, and iron intakes, respectively, were below RDA. The CRT analysis indicated that vitamin B12, iron, and folate intakes are important factors related to anemia in girls associated with age, locality, food consumption patterns, and physical activity levels, while iron and folate intakes were significant factors related to anemia in boys associated with the place of residence and the educational level of their mothers. The deployment of clustering and classification techniques for identifying the association between anemia and nutritional factors might facilitate the development of nutritional anemia prevention and intervention programs that will improve the health and wellbeing of schoolchildren.

## Introduction

Anemia is a worldwide public health concern, with 24.8% of the global population suffering from anemia (1). It is associated with many diseases that affect all the age groups (1–3). The effects of the lack of oxygen are particularly troublesome in children, as that may affect their physical growth and cognitive development (4–8). There are many causes of this disease, with nutritional inadequacies being a primary factor contributing to anemia (9–11). Various studies have associated certain nutritional insufficiencies with this disease. Iron, vitamin B12, folic acid, vitamin A, and vitamin C are some nutrients that have been linked to its development (6, 11–16). The association between nutrient intake deficiencies and other health and cognitive development problems among schoolchildren has been investigated by several studies, indicating a strong correlation between nutrition and the health of children. Researchers have investigated the prevalence of nutrient deficiencies among medically documented patients with anemia, and it has become well known that iron deficiency is the most common cause of anemia (13, 17–19). Ensuring adequate nutrient intake requires dietary variety in food consumption, which could be lacking in children age group (20–25).

Other risk factors linked to the disease include children living conditions. Goswami et al. is one of the many researchers who found that the place of residence is a major factor associated with anemia, where rural children were more anemic than urban children (26). Other researchers have also established the linkage of parental educational level with this entity (26–28). It has become clear that, as many factors interact in the development of anemia, there is a need for an in-depth study of this complex interaction, one beyond prevalence and associations.

Investigators have used many statistical methods in discerning the interaction of sociodemographic and health-related risk factors resulting in anemia. Furthermore, data mining and machine learning techniques for clustering and classifications have been improved as effective tools in the clustering and classification of risk factors associated with the health of children (29, 30). The K-means, Decision Tree, Chi-square automatic interaction detection (CHAID), the K-Nearest Neighbors' algorithm (K-NN), and Classification and Regression Trees (CRT) clustering and classification models have been used by many research studies for predicting and identifying nutrition, lifestyle, and health diseases, such as obesity, diabetes, and anemia (31–34). In medical research, several ML clustering and classification, such as support vector machine, artificial neural network, and random forest techniques, are used for improving early detection and diagnosis of diseases (29, 30, 35–38). Particularly in anemia, M. Visser et al. have employed factor analysis for the determination of the nutrient pattern that is most associated with anemia, accounting for differences between provinces (14). On the other hand, Sow et al. employed machine learning algorithms in the identification of associated sociodemographic risk factors for anemia development (38). A study in China employed the CHAID decision tree analysis for the identification of infant anemia-related risk factors (31).

To the best of our knowledge, few studies have employed cluster analysis for the classification of hemoglobin levels among a certain population. The present study is a part of the national nutritional formative research among schoolchildren aimed at assessing children's health, nutrition, and mental health. The main aim of this study was to derive dietary patterns related to anemia in schoolchildren and to investigate the association between hemoglobin, vitamin B12, iron, and folate intakes with other sociodemographic variables, and health-related risk factors among Palestinian schoolchildren. We used the K-means and CRT machine learning techniques in identifying schoolchildren clusters based on hemoglobin data and finding the association patterns through the classification of the associated risk factors.

## Materials and Methods

### Participants and Data Collection

This study utilizes data from the Health Behaviour in School-aged Children (HBSC) survey conducted by Al-Quds University and the Ministry of Health in the year 2013–2014, which aims to assess the nutritional, physical, and psychological health of Palestinian schoolchildren (34, 39, 40). The study sample included children aged 10–17 years who were enrolled in public and the United Nations Relief and Works Agency (UNRWA) for Palestinian refugee schools. A representative and clustered random sample of 5,000 students was selected from 100 schools in the West Bank and weighted for grade and age. Only the relevant variables and available data were used for this study; therefore, a final sample of 4,762 students was used for data analysis. Data collection included blood sampling, sociodemographic data, health-related practices, and 24-h dietary recalls. For hemoglobin acquisition, data were collected using the Hemoglobin (Hb) test using the HemoCue Hb 201+ analyzer. On-site 50 μl of non-fasting venous whole blood was collected for all subjects for complete blood count (CBC) analysis *via* nurses and doctors registered in the Ministry of Health on schools' grounds. The total Hb concentration was calculated to the nearest 0.1 g/dl. The anthropometric data were collected by the digital floor scale for measuring students' weights in kg and the portable stadiometer for measuring the height in cm. Prior to data collection, all parents or caregivers signed an informed consent form. The study received ethical approval from the Ministry of Education and Al-Quds University Institutional Review Board (IRB approval number: 05-Aug-2013-12/10).

### Hematological Parameter

In this study, we acquired hemoglobin levels as an indicator of anemia. The use of hemoglobin as a single anemia parameter has been applied in various research studies (10, 38, 41). In this study, the K-means clustering method was used for identifying the hemoglobin groups (42). Using the K-means clustering method, two different hemoglobin clusters were identified. The first cluster includes the participants with average hemoglobin of <12 g/dl, the second cluster includes those ≥12 g/dl.

### Health-Related Risk Factors

To measure nutrient intake, we used the 24-h food recall method. The tool gives a detailed insight into food consumption over a 24-h period (43–45). The food frequency scale was developed using the eight food items scale, which was categorized based on similarity in the nutrient profile (46). These categories were as follows: (1) vegetables; (2) fruits; (3) milk and other dairy products; (4) sweets and chocolate; (5) soft drinks; (7) beverages (juices and sugar); and (8) energy drinks. Response categories were (1) never, (2) 1–2 times a week, (3) 3–4 times a week, and (4) 5–7 times a week (almost daily). The food consumption quantities were identified using the recipes from the consumption weight book developed by ANAHRI (47). The food intake was entered and analyzed using Nutribase Professional V.9, the United States Department of Agriculture (USDA), and the Palestinian Food Recipes databases (47, 48). As studies have shown that there is an association between certain nutrients with anemia, we have chosen vitamin B12, iron, and folate as key nutrients for the analysis of and association with hemoglobin clusters. The nutrient values were analyzed and compared with the USDA Recommended Dietary Allowance (RDA) values according to the ages of participants (49).

As for health-related nutritional practices, we have used the food frequency questionnaire to classify the practices of students into (healthy and unhealthy) food consumption patterns. The indicated frequency of consumption was obtained over a week's duration (1) never, (2) 1–2 times a week, (3) 3–4 times a week, and (4) 5–7 times a week (almost daily).

The healthy group included participants who were in the top 2 quartiles (+50 percentile) of intake of fruits and vegetables (indicated as the indicators of a healthy diet by the WHO) (50) and milk and had indicated that they did not eat any unhealthy item. The unhealthy group included participants who had indicated that they did not eat any healthy nutritional items and were in the top 2 quartiles of the frequency of eating sweets, soft drinks, sugary juices, and energy drinks, respectively. The score for each variable was classified into yes and no values according to quartile classification.

Taking into consideration the numerous non-dietary risk factors that may be associated with anemia, we have included data regarding the general health status of the participants. Anthropometric measurements, such as height, weight, and neck circumference, were measured by school nurses, and the body mass index [BMI = weight/(height^2^)] of each participant was calculated. The BMI was classified into 4 categories based on the WHO cutoff values for interpretation of BMI in 5–19-year-old children (51). Furthermore, the students were asked about their physical activity during the school days and excluding weekends. The following questions comprised the frequency of physical activities over a week duration: (1) In the last week, how many days were you physically active for more than 60 min? (2) In the last week, how many hours were you playing sports outside school? (3) In the last week, how many hours do you exercise per week? These responses were summed, and the final score was classified according to its quartiles into low (1st and 2nd quartiles), moderate (3rd quartile), and high physical activity (4th quartile), respectively. In addition, as leisure time activities were accounted for in the questionnaire, the respondents were asked about their screen behavior over school days in the past week. The questions included: (1) how many hours do you spend watching TV? (2) how many hours do you play video games? (3) how many hours do you spend using the internet? Again, the sum of these questions was used to generate a leisure time activity scale, and the quartiles were used to classify the outcome into low (1st and 2nd quartiles), moderate (3rd quartile), and high (4th quartile) leisure time activities. Moreover, students were asked about their smoking practices, and the responses were yes or no to smoking cigarettes and/or nargileh, respectively. Furthermore, as anemia could result from a multitude of chronic disease processes, we asked the students and their parents about any known chronic illnesses, in addition to checking the available school records on the chronic illnesses of students, and we ultimately excluded those students from this study.

### Statistical Analysis

We employed descriptive statistics, the K-means clustering method, and the CRT technique (52). The statistical analysis was conducted using IBM Statistical Package for Social Science V21. The K-means clustering method is a type of statistical analysis that classifies the sample population into homogenous groups with different characteristics using a specific variable as the comparison criterion (42). The K-means clustering method defines the segments of a dataset and assigns each observation into a specific cluster, the algorithm identifies the smallest variation within each cluster. A non-hierarchical K-means clustering method was used to produce two hemoglobin clusters, with the random seed and 10 iterations to refine and optimize the classifications. The final clusters were selected based on interpretability and the percentage of participants in each cluster. The sociodemographic and nutrition variables were analyzed for these clusters. Chi-squared and univariate analysis tests were used to assess the differences between categorical data. Continuous data were assessed for normality and, if required, normalized with natural log transformation. Of the available decision tree models, we chose the CRT model for the identification of potential risk factors for childhood anemia, relating to each relevant nutrient of choice (vitamin B12, iron, and folate). Cases in each subgroup are further classified by the second most significant predictor. The analysis continues until the last significant risk factor is identified.

## Results

### Demographics and Health-Related Risk Factors

The results of the demographic characteristics and health-related risk factors are depicted in Table 1. The study sample consists of 66.9% girls and 33.1% boys, 72.6% aged 10–13 years, and 27.4% aged 14–17 years. The students were mostly from urban habitats (42.8%), but 34.6% were from rural habitats and the rest were from refugee camps. When asked about their family income, almost 45% of the participants reported they had low family income compared with the minimum wages in the West Bank, and only 19.2% had high income, respectively. Additionally, students were asked about the level of their parents' education. Over two-thirds reported that their parents have an education level of secondary school and above, 60.4% for fathers and 61.4% for mothers, respectively. The results of nutritional status assessment indicated that almost 80% were found to have normal BMI levels, while 5.1% exhibited obesity as per the BMI scale. Of the study participants, 50% reported healthy food consumption and 68% reported unhealthy food consumption. Most students reported medium or high levels of physical activity (74.3%), as well as leisure time activities (73.9%), respectively. Furthermore, about 16.5% of students were smoking cigarettes or nargileh.

**Table 1:** Demographic variables of the study sample (*n* = 4,762).

				**Hemoglobin**
		***n***	**%**	**Mean ±SD**
Gender	Boys	1,575	33.1	13.1 ± 1.35
	Girls	3,187	66.9^**^	12.9 ± 1.27
Age	10–13 years	3,455	72.6^**^	12.9 ± 1.23
	14–17 years	1,307	27.4	13.07 ± 1.47
Locality	Urban	2,037	42.8^**^	13.1 ± 1.26
	Rural	1,650	34.6	12.8 ± 1.29
	Camp	1,075	22.6	12.9 ± 1.38
Family income	L	2,122	44.6	12.9 ± 1.3
	M	1,724	36.2	13 ± 1.3
	H	916	19.2	13 ± 1.32
Father's education	≤ Secondary	1,887	39.6	13 ± 1.3
	>Secondary	2,875	60.4	12.9 ± 1.31
Mother's education	≤ Secondary	1,838	38.6	12.9 ± 1.3
	>Secondary	2,924	61.4	13 ± 1.31
BMI	Underweight	234	4.9	13 ± 1.29
	Normal	3,807	79.9	12.9 ± 1.42
	Overweight	480	10.1	12.8 ± 1.34
	Obese	241	5.1	12.9 ± 1.27
Healthy food consumption	No	2,019	42.4	13 ± 1.33
	Yes	2,743	57.6	13 ± 1.31
Unhealthy food consumption	No	1,506	31.6	13 ± 1.3
	Yes	3,256	68.4	13 ± 1.3
Smoking	No	3,977	83.5	13 ± 1.35
	Yes	785	16.5	12.9 ± 1.3
Physical activity	L	1,223	25.7	13 ± 1.29
	M	1,321	27.7	13 ± 1.32
	H	2,218	46.6	12.9 ± 1.31
Leisure time activity	L	1,245	26.1	13 ± 1.25
	M	1,281	26.9	13 ± 1.33
	H	2,236	47.0	13 ± 1.31

*N, number of study samples; SD, standard deviation; L, low; M, Medium; H, high*.

** *p < 0.001*.

Table 1 depicts the mean and standard deviation (SD) of hemoglobin values for each demographic variable category. There were statistically significant differences in hemoglobin values per age, gender, and locality (*p 0*.000, 0.000, and 0.000, respectively). The mean hemoglobin values were higher in boys than girls (13.1 and 12.9, respectively), and in the older age group (13.07). Moreover, the mean hemoglobin was higher in urban than rural and camp residents (13.1, 12.8, and 12.9), respectively.

### Overall Nutritional Profile

The macronutrient and micronutrients intake analysis of 24-h food recall data is reported in Table 2. All intakes differed significantly by age and gender (*p* < 0.05). The results indicated that the mean energy intake was higher in boys than girls across both age groups (2494.2, 2570.6) and (2101, 1936.7), respectively. In general, boys had a higher nutrient intake of carbohydrates, proteins, fat, cholesterol, vitamin B12, iron, and folate than girls.

**Table 2:** Mean (± SD) nutrient intake per 24-h by age and gender.

	**Age (years)**			
	**10–13**		**14–17**	
	**Boys**	**Girls**	**Boys**	**Girls**
Energy (kcal)	2,494.2 ± 799^**^	2,101 ± 743.4	2,570.6 ± 859.4^**^	1,936.7 ± 731.3
Carbohydrates (g)	347.9 ± 120.1^**^	297.1 ± 105.9	362.9 ± 128.1^**^	276.3 ± 110.8
Protein (g)	88.8 ± 36.4^**^	72 ± 32.5	89.9 ± 38.4^**^	65.1 ± 30
Fat (g)	86.4 ± 36.8^**^	72.6 ± 32.9	88 ± 39.8^**^	66.6 ± 31
Cholesterol (mg)	256.2 ± 224.2^**^	197.1 ± 190.8	239.2 ± 224.7^**^	160.9 ± 154.3
VitB12 (mcg)	2.5 ± 2.7^*^	2 ± 2.3	2.7 ± 3^*^	1.7 ± 2
Iron (mg)	14.5 ± 6.8^**^	13.2 ± 6.9	14.1 ± 6.8^**^	11.7 ± 6.6
Folate (mcg)	298 ± 195.6^**^	250.9 ± 182	288.4 ± 181.8^**^	234.5 ± 171.5

*SD, standard deviation; kcal, kilocalories; g, grams, mcg; micrograms; Vit, vitamin*.

* *p < 0.05*.

** *p < 0.001*.

Table 3 reports the percentage distribution of students' vitamin B12, iron, and folate intake according to the RDA as nutritional anemia indicators. All intakes differed significantly by age and gender (*p* < 0.05). Both boys and girls had vitamin B12 intake lower than the RDA across both age groups, with a higher percentage in girls (52.2 and 56.8% for boys, and 60.8 and 74% for girls, respectively). Iron intake was above the RDA for all boys (84.9 and 67.7%), and younger (10–13 years) girls (71.9%), respectively. However, 65.7% of older girls (14–17 years) had iron intake below RDA. Folate intake was below RDA for boys and girls across all age groups, with a lower intake for boys (61.1 and 77.6% for boys; 71.9 and 86.4% for girls), respectively.

**Table 3:** Nutrient intake levels according to the Recommended Dietary Allowance (RDA) per age and gender.

		**Age (years)**			
		**10–13**		**14–17**	
		**Boys**	**Girls**	**Boys**	**Girls**
		***n*** **(%)**			
VitB12 (mcg)	< RDA	599 (52.5)	1,407 (60.8)^**^	246 (56.8)	647 (74)^**^
	≥RDA	543 (47.5)	906 (39.2)	187 (43.2)	227 (26)
Iron (mcg)	< RDA	172 (15.1)	556 (24)	140 (32.3)	574 (65.7)
	≥RDA	970 (84.9)^**^	1,757 (76)	293 (67.7)^**^	300 (34.3)
Folate (mcg)	< RDA	698 (61.1)	1,662 (71.9)^**^	336 (77.6)	755 (86.4)^**^
	≥RDA	444 (38.9)	651(28.1)	97 (22.4)	119 (13.6)

*n, number of study sample; SD, standard deviation; mcg, micrograms; Vit, vitamin*.

** *p < 0.001*.

### K-Means Cluster Analysis of Hemoglobin

Table 4 shows the results of the K-means clustering of the hemoglobin values of the study participants. The clustering technique resulted in two distinct hemoglobin clusters, with statistically different average hemoglobin values of 11.85 and 13.9 g/dl (*p* < 0.05). The clusters were identified as low and high (46.4 and 53.6%) in reference to the WHO criteria for anemia definition, for both genders (1). There were significant differences in the distribution of children across hemoglobin clusters by age, gender, and locality. The high hemoglobin cluster included 915 boys and 1,636 girls. A higher percentage of students from both age groups were classified into the high hemoglobin cluster (52.2 and 57.2%). Furthermore, more students from urban and camps residences were classified into the high hemoglobin cluster (57.5 and 51.8%), while 50.8% of rural residents were in the low hemoglobin cluster.

**Table 4:** Distribution of study sample by hemoglobin clusters.

		**Hemoglobin clusters**	
		**Low (<12g/dl)**		**High (≥12g/dl)**
		***n*** **(row%)**		
Total sample		2,211 (46.4)		2,551 (53.6)
Gender	Boys	660 (41.9)		915 (58.1)^**^
	Girls	1,551 (48.7)^**^		1,636 (51.3)
Age (years)	10–13	1,650 (47.8)^**^		1,802 (52.2)
	14–17	559 (42.8)		746 (57.2)^**^
Locality	Urban	865 (42.5)		1,172 (57.5)^**^
	Rural	828 (50.2)^**^		822 (49.8)
	Camp	518 (48.2)		557 (51.8)
			mean ± SD	
Hemoglobin level		11.85 ± 0.8		13.9 ± 0.8^**^

*n, number of study sample; SD, standard deviation*.

** *p < 0.001*.

### Nutrition Profile per Hemoglobin Clusters

Table 5 shows the mean energy and main nutrients among the hemoglobin clusters. The mean energy, carbohydrates, vitamin B12, and fat are roughly equal among the low and high hemoglobin clusters (2,207.7, 2,208; 311.4, 311.5; 2.1, 2.2; and 76.3, 76.1). The high hemoglobin cluster had higher mean protein, iron, and folate intake (76.7, 17.8, and 263.6). These differences, however, were not found to be statistically significant across hemoglobin groups.

**Table 5:** Mean (±SD) distribution of nutrient intake by hemoglobin clusters.

**Nutrient (unit)**	**Hemoglobin clusters**	
	**Low (<12g/dl)**	**High (≥12g/dl)**
Energy (kcal)	2,207.7 ± 784.9	2,208 ± 809.6
Carbohydrates (g)	311.4 ± 114.7	311.5 ± 117.7
Protein (g)	75.9 ± 34.2	76.7 ± 35.5
Fat (g)	76.3 ± 34.2	76.1 ± 35.8
VitB12 (mcg)	2.1 ± 2.4	2.2 ± 2.5
Iron (mg)	17.1 ± 13	17.8 ± 13.5
Folate (mcg)	261.4 ± 188.9	263.6 ± 181.6

*SD, standard deviation; kcal, kilocalories; g, grams; mcg, micrograms; Vit, vitamin*.

### CRT Analysis of Anemia Related Risk Factors

**Figure 1:**
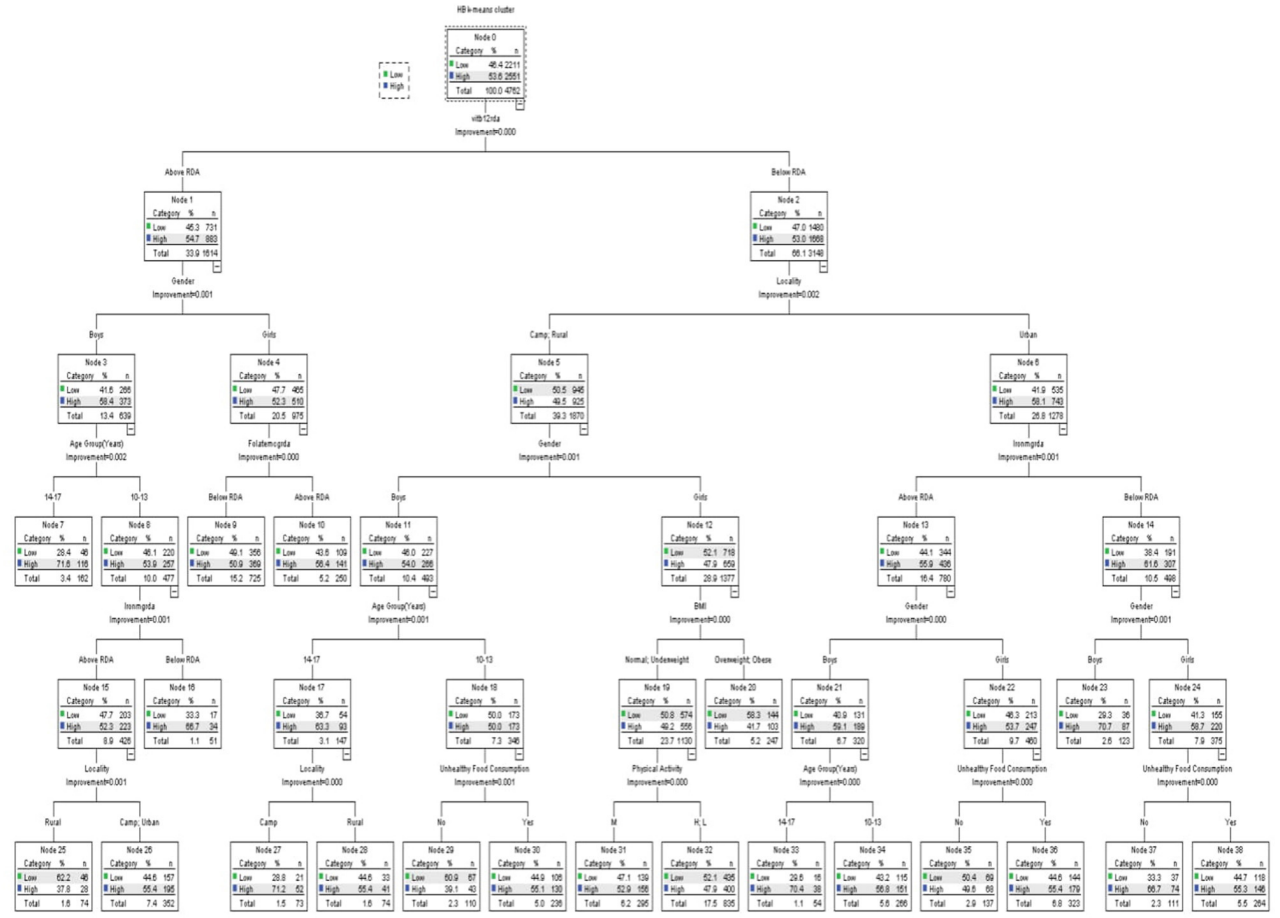
Classification and regression decision tree (CRT) analysis of anemia-associated risk factors as per vitamin B12 intake.

**Figure 2:**
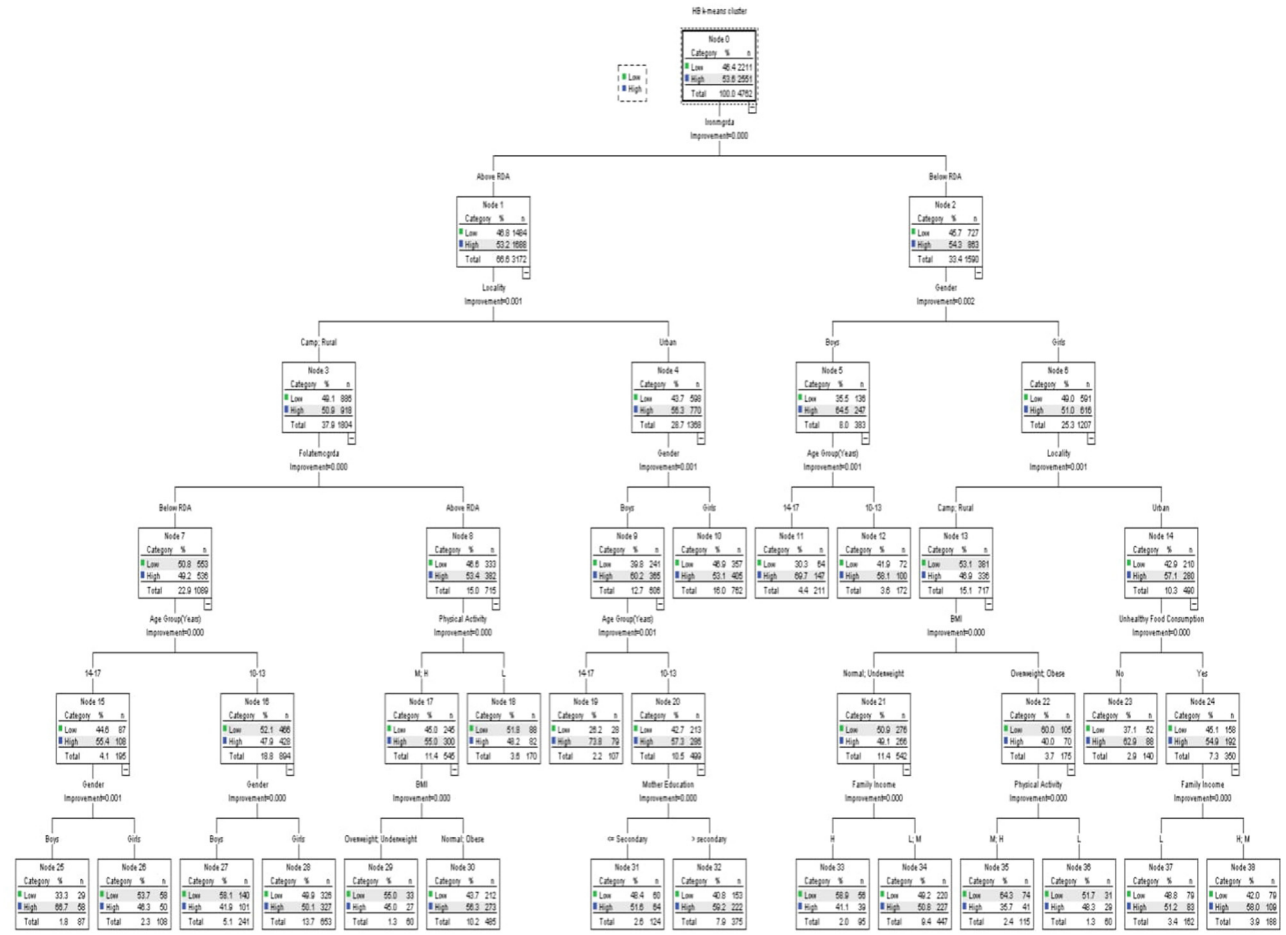
Classification and regression decision tree (CRT) analysis of anemia-associated risk factors as per iron intake.

**Figure 3:**
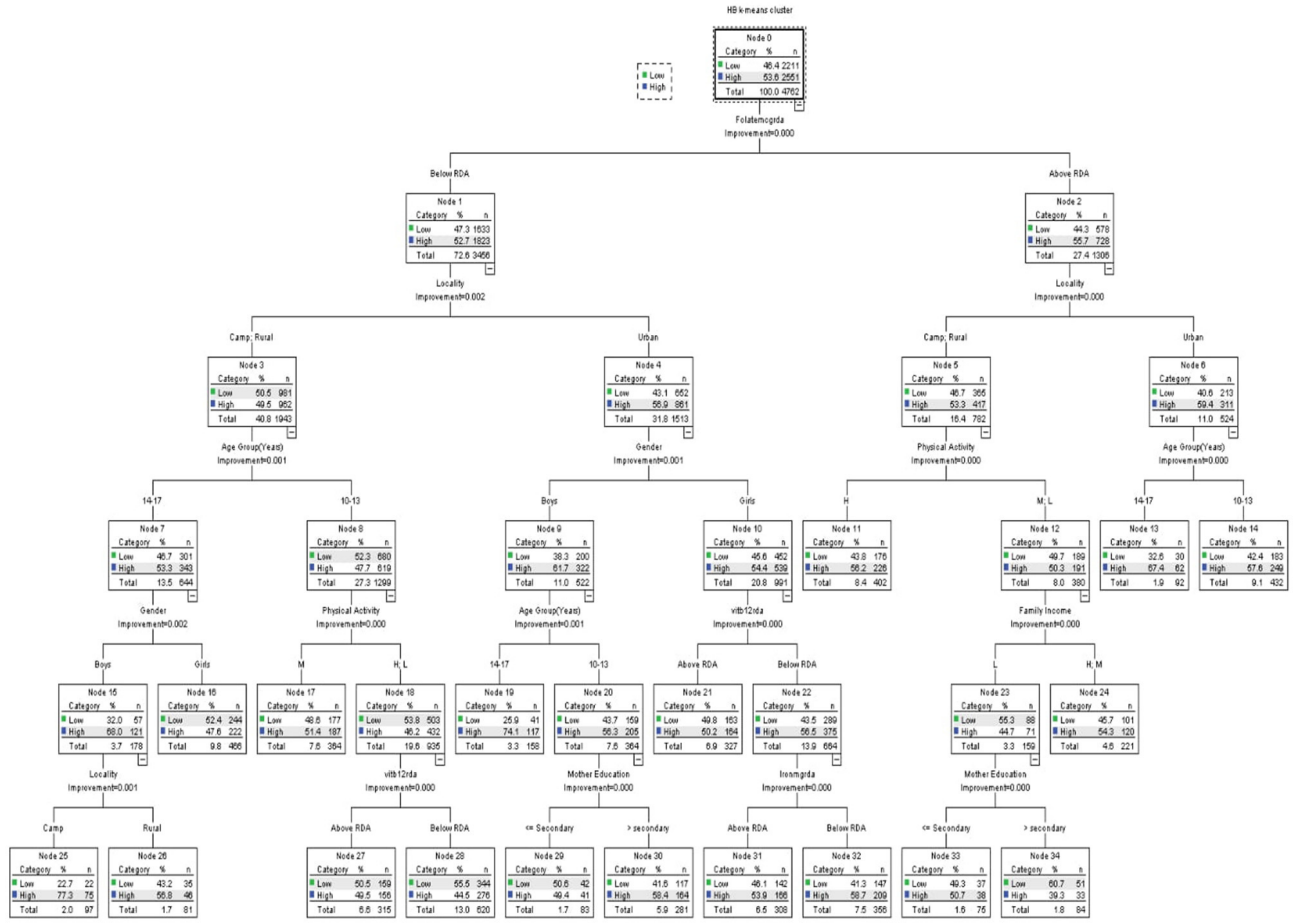
Classification and regression decision tree (CRT) analysis of anemia-associated risk factors as per folate intake.

The CRT classification technique has been used for classifying the hemoglobin clusters by schoolchildren's nutritional intake (vitamin B12, iron, and folate). Table 6 shows the variables included in the CRT classification analysis. Figures 1–3 show the CRT analysis. Each node contains three statistical values (category, %, *n*) in addition to node number, category stands for vitamin B12, iron, and folate intake, *n* stands for low and high hemoglobin clusters in this category, and % is the percentage of students in each cluster.

**Table 6:** Classification tree variables' description.

**Variable name**	**Description**	**Values**
Gender	Gender	Boys, girls
Age	Age	Age (10–17 years)
FAS	Economic status	Low, medium, high
FatherEdu	Father education	≤ Secondary, >Secondary
MotherEdu	Mother education	≤ Secondary,>Secondary
HealthConsump	Healthy food consumption	Low, moderate, high
UnhealthyConsump	Unhealthy food consumption	Low, moderate, high
BMI	Body mass index	Underweight, normal, overweight, or obese
Smoking	Tobacco risk	Yes, no
PA	Physical activity	Low, moderate, high
Calories	Energy in kilocalories	Mean
Carbs_g	Carbohydrates in grams	Mean
Protein_g	Protein in grams	Mean
Fatg	Fat in grams	Mean
Vitb12rda	Vitamin B12 intake per recommended dietary allowance (RDA)	Below RDA, above RDA
FolatemcgRDA	Folate intake per recommended dietary allowance (RDA)	Below RDA, above RDA
Ironmgrda	Iron intake per recommended dietary allowance (RDA)	Below RDA, above RDA

The CRT classification of hemoglobin in reference to vitamin B12 is shown in Figure 1, the classification tree includes a total of 39 nodes with 20 terminal nodes. In each category, the population of hemoglobin clusters differs significantly (all *p* < 0.05). The estimated error of risk in the model is 0.439, and the standard error (SE) is 0.007. The vitamin B12 intake was classified into below and above RDA values, the below RDA group was classified by locality, iron, gender, unhealthy food consumption, age, BMI, and physical activity, while the above RDA group was classified by gender, folate, age, iron, and locality. The below RDA node by locality was classified into two groups, urban group and camp and rural group, and the urban group was classified by iron intake (below and above RDA); the below group was classified by gender and unhealthy food consumption. Girls reported a higher percentage of low hemoglobin levels that were significant with unhealthy food consumption. The camp and rural group was further classified by gender. Boys were classified by age group, whereas girls were classified by BMI and physical activity. Other variables did not reach a significance of 0.05 and were not included in the model, such as father's and mother's education, healthy food consumption, and leisure time activity.

Figure 2 shows the hemoglobin clusters' classification per iron intake. The model includes a total of 39 nodes with 20 terminal nodes. In each category, the population of hemoglobin clusters differs significantly (all *p* < 0.05). The estimated error of risk in the model is 0.441, and the SE is 0.007.

The iron intake was classified into below and above RDA groups; the below group was classified by gender, locality, age group, unhealthy food consumption, BMI, physical activity, and family income. The above group was classified by locality, gender, folate intake, age group, physical activity, BMI, and mother education. The below RDA group was further classified by gender; the boys' group were associated with age, while the girls' group were further classified by locality, in which the urban residents group was associated with unhealthy food consumption, whereas those who had high unhealthy food consumption were classified by their family income. However, camp and rural residents were classified by their BMI level, and those who are overweight or obese were further classified by their physical activity. Those who have normal BMI or are underweight were classified by family income.

On the other hand, the above RDA group was classified first by locality (camp and rural residents and urban residents). Among the camp and rural residents group, the hemoglobin level was affected by folate intake. In the left branch, those who had low folate intake were classified by gender and then by age. In students who had high folate intake, physical activity was a significant factor, split into low, medium, and high levels, which were classified by the BMI level. In the latter group, those in the urban residence group were classified by gender, and boys were classified by age, and younger students (10–13-year-old) were further classified by the education level of their mothers, respectively.

Figure 3 demonstrates the hemoglobin clusters' classification per folate intake. The model includes a total of 35 nodes with 18 terminal nodes. In each category, the population of hemoglobin clusters differs significantly (all *p* < 0.05). The estimated error of risk in the model is 0.441, and the SE is 0.007.

The folate intake was classified into below and above RDA values, the below RDA group was classified by locality, age group, physical activity, family income, and mother's education. The above RDA group was classified by locality, gender, age group, vitamin B12 intake, iron intake, and mother's education. Both folate intakes above and below RDA values were first classified by locality. On the left branch of the tree, the camp and rural residents were classified per age. Older students (14–17 years) were further classified by gender; boys were classified by their residence in camp and rural areas. Younger students (10–13 years) were classified by physical activity; those who had either low or high levels of physical activity were further classified by their vitamin B12 intake. Residents of the urban were first categorized by gender. Boys were classified by their age, where the education of a mother was a significant factor in younger boys. On the other hand, girls were first classified per their vitamin B12 intake; those who had vitamin B12 intake below RDA were further classified according to their iron intake. On the right branch of the tree, those who had higher folate intake and lived in camps or rural areas were classified based on their physical activity levels. Those who had either low or medium levels were affected by their family income. Furthermore, those with low family income were classified by the education level of their mothers. Those who lived in urban residences were only classified by their age.

## Discussion

Anemia remains a major health concern among schoolchildren (6). Almost half of the sample were found to have mean hemoglobin levels below the WHO cutoff value for anemia (1). The results of the present study indicate that there is an interlinked association between nutritional intake and sociodemographic factors in the development of the disease, which is consistent with other studies (11, 18, 41). The sole deficiency of certain nutrients is rarely found as a single nutritional anemia associated factor. The CRT analysis partitioned the sample's hemoglobin clustered groups into homogeneous subgroups, taking vitamin B12, iron, and folate intakes as key splitting variables. The results indicate that two-thirds of hemoglobin clusters were found to have a vitamin B12 intake below the RDA level and were significantly associated with the place of residence factor. This result is consistent with studies that associate living place and anemia among children (26, 41, 53). Interestingly, our results indicate that weight and physical activity are significant anemia-related factors in girls living in camps and rural areas. Whereas, in urban areas, iron intake and unhealthy food consumption were found to be significant anemia-related factors, which could be explained by the differences in lifestyle and eating habits between these residences. Furthermore, girls in camps and rural areas spend more indoor time than those in urban areas, which might lead to limited access to physical activities and higher obesity rates furthermore, this finding is consistent with other studies which indicated that obesity is associated with vitamin B12 deficiency and iron deficiency, therefore possible nutritional anemia (54–57). The coexistence of these nutrients' deficiencies has been observed in other studies. Ahmed et al. have demonstrated the prevalence of coexisting vitamin B12 and iron deficiencies among adolescent girls, albeit in rural rather than urban areas as found in our study (58). In boys, however, we have observed that the anemia differs significantly across the urban and non-urban areas by age, where younger boys have the higher rates of anemia. This finding is inconsistent across studies. Research carried out in Ethiopia, for example, has found that anemia levels are lower in 10–13-year-old boys than older adolescents (59); however, other studies have demonstrated that anemia is higher among early adolescent years (10–13 years) compared with the late adolescent period (60). Another study in Nepal concluded that the anemia level in older boys and girls (15–19 years) is higher than the anemia level in younger children (61).

Even those who have an adequate vitamin B12 intake can still be affected by other variables. It seems that, in this group, iron intake is a significant factor in young boys, while the hemoglobin levels of girls might be more affected by folate intake. This emphasizes the need for a holistic approach to nutrient intake enhancement when developing policies to prevent nutritional anemia.

On the other hand, when we classified the anemic status with iron intake, it seems that lower iron intake is higher in girls. Furthermore, anemia in girls residing in urban areas is associated with food consumption and family income, whereas in camps and rural areas, it seems that obesity and lower physical activity correspond with the higher rates of anemia. In boys, age again is a major factor whatever their iron intake may be. Of note, folate intake seems to affect both boys and girls in non-urban areas with adequate iron intake; however, it is more significant in younger boys and older girls, respectively. This result coincides with the literature, which has found that average folate intake in girls differs by age (15) and between boys and girls, especially in 13–15-year-old children (62).

When we classified hemoglobin clusters by folate intake, there were differences by locality across all levels of folate intake. Younger children living in camps and rural areas who had a lower intake of folate are affected by lower physical activity and lower vitamin B12 intake. However, the hemoglobin levels of older children in the same conditions are affected by age and gender, where anemia is higher among girls. This may indicate that focusing on the folate levels of girls in camps and rural areas might significantly affect their anemic status. On examining urbanite children with folate intake lower than the RDA, vitamin B12 and iron intake are important factors, however, young boys are particularly affected by the education of their mothers. Unexpectedly, in this group,the hemoglobin level is higher in boys whose mothers have a lower education. This could be explained by the fact that mothers with lower education are most likely housewives and spend more time with their children and family, which might contribute to the better care of children's health and nutrition. This finding needs further investigation, as the educational status of mothers is found to be positively correlated with the lower odds of anemia across children in numerous studies (26, 63, 64).

The K-means and CRT clustering and classification techniques showed a different pattern of nutritional deficiencies in reference to the hemoglobin results of schoolchildren. The classification by vitamin B12 is associated with locality, iron, gender, unhealthy food consumption, age, BMI, and physical activity. The classification by iron intake is associated with gender, locality, age group, unhealthy food consumption, BMI, physical activity, and family income. The classification by folate intake is associated with locality, age, gender, physical activity, vitamin B12, locality, mother's education, and iron intake.

The findings of this study have considerable implications for researchers and policymakers to reduce the prevalence of nutritional anemia. This study supports the implementation of data mining and machine learning techniques in developing AI solutions for the development of patient-specific anemia prevention and intervention programs. As indicated, there are several sociodemographic gaps that have to be taken into consideration when designing interventional strategies to improve nutritional deficiencies. Overall, it seems that it is rather important to address the vitamin B12, iron, and folate intake in girls, while accounting for age, locality, food consumption patterns, and physical activity levels, and focus on iron and folate intake in young boys with consideration to the place of residence. However, there are some limitations to our study, namely, the use of a single 24-h recall interview, and the nutrient was calculated from food intake rather than blood biomarkers. Furthermore, the study sample only included the schoolchildren age group and did not include the younger age groups (<10 years). Hopefully, the findings of this study might aid policymakers in signifying the population groups requiring priority attention, what strategy to choose, and where to allocate public resources, to customize intervention plans based on the most significant factors involved in nutritional anemia occurrence.

## Data Availability Statement

The raw data supporting the conclusions of this article will be made available by the authors, without undue reservation.

## Ethics Statement

The studies involving human participants were reviewed and approved by Ministry of Education and Al-Quds University Institutional Review Board (IRB). Written informed consent to participate in this study was provided by the participants' legal guardian/next of kin.

## Author Contributions

RQ and DA: conceptualization, methodology, data curation, formal analysis, validation, writing—original draft preparation, and review and editing. All authors contributed to the article and approved the submitted version.

## Funding

The work included in this study is part of the wider project Determinants of Cognitive Development in Deprived Environments: evidence from the West Bank funded by the German Research Foundation (DFG) under Grant Number JU 2769/2.

## Conflict of Interest

The authors declare that the research was conducted in the absence of any commercial or financial relationships that could be construed as a potential conflict of interest.

## Publisher's Note

All claims expressed in this article are solely those of the authors and do not necessarily represent those of their affiliated organizations, or those of the publisher, the editors and the reviewers. Any product that may be evaluated in this article, or claim that may be made by its manufacturer, is not guaranteed or endorsed by the publisher.
